# *Lemna gibba* Clones Show Differences in Phenotypic Responses to the Light Environment

**DOI:** 10.3390/plants14182840

**Published:** 2025-09-11

**Authors:** Pham Thi Hong Xuan, Raja Amri, Nguyen Phuong Bach, Muhammad Irfan, Manuela Bog, Klaus J. Appenroth, K. Sowjanya Sree, Marcel A. K. Jansen, Sándor Szabó, Ilona Mészáros, Viktor Oláh

**Affiliations:** 1 Department of Botany, Faculty of Science and Technology, University of Debrecen, Egyetem sqr. 1, H-4032 Debrecen, Hungaryamriraja05@gmail.com (R.A.); immeszaros@unideb.hu (I.M.); 2 Doctoral School of Biological Sciences, Szent István Campus, Hungarian University of Agriculture and Life Sciences, Páter Károly st. 1., H-2100 Gödöllő, Hungary; 3 School of Biological, Earth and Environmental Science, University College Cork, Distillery Fields, North Mall, T23N73K Cork, Ireland; mirfan@ucc.ie (M.I.); m.jansen@ucc.ie (M.A.K.J.); 4 Institute of Botany and Landscape Ecology, University of Greifswald, Soldmannstr. 15, 17489 Greifswald, Germany; manuela.bog@uni-greifswald.de; 5 Matthias Schleiden Institute—Plant Physiology, University of Jena, 07743 Jena, Germany; klaus.appenroth@uni-jena.de; 6 School of Biotechnology, Institute of Science, Banaras Hindu University, Varanasi 221005, India; kssree9@bhu.ac.in; 7 Department of Biology, Institute of Environmental and Natural Sciences, University of Nyíregyháza, H-4401 Nyiregyhaza, Hungary; szabo.sandor@nye.hu

**Keywords:** duckweed, frond, light acclimation, biodiversity, photosynthesis, photosynthetic pigments, chlorophyll fluorescence

## Abstract

Duckweeds are aquatic plants with a worldwide distribution that can thrive under very contrasting ambient conditions due to their diversity and high phenotypic plasticity. In this study, we analyzed and compared the responses of four clones (i.e., distinct accessions) of *Lemna gibba* to two different light intensities. The clones represented different geographical origins and, in addition to two diploid cytotypes, included one tetraploid mutant and a triploid interspecific hybrid. We hypothesized that clonal origin had an effect on light acclimation. We studied growth, morphological (frond size and shape, mass-to-area ratio), and photosynthetic (pigment composition, chlorophyll fluorescence induction) traits of these clones to test whether light acclimation was a conserved process or whether clone-specific strategies could be found. We also analyzed frond-level photosynthetic adjustment during ontogenesis to track how light acclimation evolved in developing fronds. Our results confirmed that even clones of the same duckweed species and a hybrid of closely related species followed partially different strategies in acclimation to ambient conditions. This acclimation involved various morphological, physiological, and biochemical adjustments but happened in a very short time window at the early life stage when young, still-developing fronds could flexibly achieve an optimized phenotype. In addition to explaining the worldwide success of duckweeds in colonizing very contrasting habitats, our results also highlight the importance of approaching duckweed responses at the frond level, where the actual acclimation takes place.

## 1. Introduction

Duckweeds (Lemnaceae) form a unique free-floating plant group that has adapted to life in freshwater aquatic environments, often rapidly colonizing and even dominating lentic habitats. In the course of this adaptation, they have undergone considerable morphological reductions, resulting in a flat or globous body plan, usually termed frond, with a size in the mm-cm range [1,2,3]. This simplified frond contains all the assimilating and reproductive parts and, in the more ancient genera (*Spirodela*, *Landoltia*, and *Lemna*), also features vascular elements and adventitious roots [3,4]. Duckweeds most commonly reproduce in a vegetative way, with frond meristems forming offspring. A frond is easily capable of forming 10–15 daughter fronds during its ~1-month-long lifespan [5,6]. The newly formed fronds start to produce their own offspring while still attached to their mothers, resulting in larger clusters (colonies) of ramets. At the population scale, this strategy results in an extremely fast reproduction rate with doubling times well below 2 days under suitable conditions [7,8]. In part, because of this rapid biomass growth, commercial applications of duckweed are of increasing interest in such fields as wastewater management, biofiltration, biofuel, feed, and food production [9,10]. Optimized production of duckweed biomass, however, requires knowledge of the environmental responses of these tiny plants.

Lemnaceae are spread across wide bioclimatic ranges [11,12]. For example, common duckweed (*Lemna minor* L.) is native to most of Europe, Africa, North America, and Asia, while also thriving (as an alien, introduced species) in Australasia and South America. Thriving in habitats with contrasting environmental conditions requires high phenotypic plasticity. Indeed, duckweeds have been reported to effectively respond to extremes in nutrient and light availability, temperature, crowding, etc. [13,14,15,16]. In response to different light conditions, duckweeds can reach their inherent maximal relative growth rates (RGR) at relatively low photosynthetic photon flux densities (PPFD = 50–100 μmol m^−2^ s^−1^). They can still maintain, however, the same RGR at much higher PPFD (~1000 μmol m^−2^ s^−1^) and at daily light integrals (DLI) exceeding the longest and brightest days on Earth (86 mol m^−2^ d^−1^) [17,18]. Photosynthetic adjustment to such contrasting light environments, while the carbon sink by growth stays constant, is of key importance for the worldwide occurrence and success of duckweeds [19]. This phenotypic flexibility involves different acclimation responses, including adjustments of morphological and anatomical traits, chloroplast ultrastructure, photosynthetic pigment composition, electron transport capacity, enzymatic capacity, and photoprotective capability [20,21]. Due to the rapid ontogenetic development of fronds, however, adjustment to ambient light must happen in a short window of just a few days. Duckweed fronds develop and mature basipetally [22]. First, the apical tip of a daughter frond protrudes from the mother frond’s meristematic pocket, then it elongates and matures while the more basal parts are still in the process of differentiation. As the rest of the frond emerges from the mother frond, maturation proceeds gradually towards the basal end. A similar basipetal light acclimation pattern can be hypothesized for the photosynthetic machinery of fronds. Previous results confirmed that the photochemical efficiency was lower in developing *Lemna* fronds than in their mother fronds and increased gradually along the fronds’ longitudinal axis [23,24,25,26].

Another outcome of the wide geographic distribution and short life cycle of duckweeds is their inherent diversity with many locally adapted ecotypes [7]. In addition, as part of their natural diversity, recent studies have shown evidence of various cytotypes with different ploidy levels within many Lemnaceae species, as well as amongst interspecific hybrids [27,28,29,30]. With respect to photosynthetic processes, polyploidy can result in metabolic imbalances due to different transcript levels in the nuclear and plastid genomes, affecting the plants’ fitness [31,32,33]. Polyploidization, on the other hand, can also promote genetic variation through increasing the number of mutation events in spare gene copies and by retaining recessive mutations—irrespective of their beneficial or deleterious nature under the given conditions—in the genome [32]. This genetic diversity supports the widespread occurrence of duckweeds due to a widened spectrum of responses to fluctuating habitat conditions. To better assess and utilize this inherent diversity, several duckweed accession (hereafter termed as “clones”) collections have been established worldwide as axenic cultures [34]. Practical applications can especially profit from the genetic diversity of duckweed clones. For example, better utilization of habitat resources by a particular clone, or its higher tolerance to stressors, can improve the performance of duckweed-based systems. Acclimation to ambient light, as one of the key drivers in plant fitness, has been extensively studied in duckweeds. Most studies, however, reported light responses of single clones and generalized them to the studied species (e.g., [19,20]). Recent evidence, in contrast, underlines that intraspecific diversity—including, e.g., different ploidy levels and geographic origins—should, in fact, be taken into account when interpreting duckweed responses [3,15,28,31]. Genetic diversity can become highly relevant in another application field too. Duckweeds are popular models in plant biology and ecotoxicology, but intraspecific diversity may strongly affect the observed phenotypic responses, e.g., growth rates [7,35]. Hence, it is important to gain insights into how clonal differences can shape duckweed responses to various environmental stimuli.

In the present study, we analyzed and compared responses of four *Lemna gibba* clones to different light intensities. Two clones displayed diploid cytotypes, while one clone was a triploid hybrid, and the fourth one was a tetraploid mutant. We hypothesized that these differences, clonal origin in general, and polyploidy/hybrid nature in particular, alter the plant’s ability to acclimate to light. Therefore, we compared each clone’s growth rate, frond morphology, and photosynthetic performance to address whether light acclimation was a conserved process across *L. gibba* or varied among clones, revealing clone-specific strategies.

## 2. Materials and Methods

### 2.1. Stock Culturing and Experimental Conditions

The duckweed clones used in this study are listed in Table 1, while their relative genome sizes are provided in Figure S1 and Table S1. Two clones were isolated in Hungary, while one clone (#9602) was a spontaneous tetraploid mutant of the widely used clone *L. gibba* 7796, traditionally known as clone G3. A detailed description of this lineage was provided by Sarin et al. [31]. The 4th clone was originally isolated in Ireland, and, based on its genome size, it was identified as a triploid hybrid of *L. gibba* and *Lemna minor* (i.e., *L.* × *mediterranea*), with a subgenome composition of GGM (Figure S1 and Table S1).

**Scheme 1:**
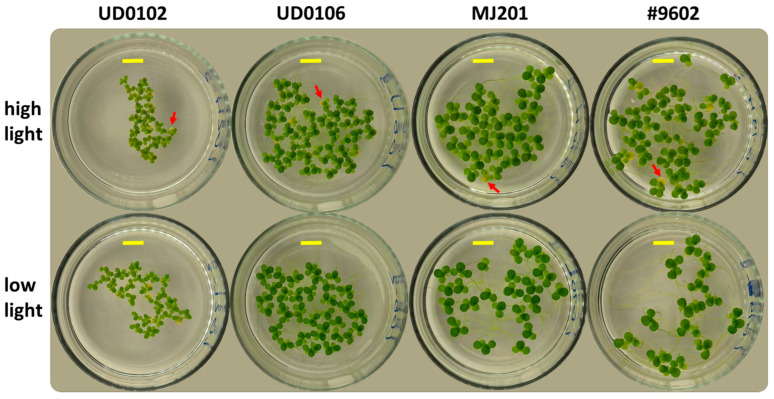
Cultures of the four *L. gibba* clones at the end of the 7-day-long growth trials under low- (100 μmol m^−2^ s^−1^, lower row) and high-light treatments (243 μmol m^−2^ s^−1^, upper row), respectively. Yellow markers denote 1 cm. Red arrows in the high-light cultures point to senescent fronds.

The axenic stock cultures were maintained in a plant growth chamber (FH-721, Taiwan Hipoint Corporation, Taiwan) under constant conditions in 300 mL conical flasks containing 100 mL autoclaved Steinberg medium (pH 6.0) prepared according to the recipe in Environment Canada [36]. Continuous irradiation (i.e., 24:0 h light/dark photoperiod) was provided by combining red (peak intensity at 657 nm), blue (peak intensity at 452 nm), and wide-spectrum white LEDs (peak intensity at 577 nm and a lower secondary peak at 467 nm, Supplementary Figure S2). Separate stock cultures were acclimated to two distinct light levels of 100 and 243 μmol m^−2^ s^−1^ PPFD (±5%), summing up to 8.64 and 21.00 mol m^−2^ d^−1^ DLI, respectively. The light intensities were recorded and averaged (*n* = 5 for either intensity) by means of an MS-100 InSight spectroradiometer (Apogee Instruments Inc., Logan, UT, USA). Although the higher light intensity was not extremely high, the two light environments resulted in distinct phenotypes in the studied *L. gibba* clones (Scheme 1). Additionally, the lower light intensity was below, while the higher one was over the DLI saturation point for growth of duckweeds and fell into the typical PPFD and DLI range of reported duckweed cultivation systems and duckweed-related studies conducted indoors [18]. Therefore, for the sake of simplicity, the two light intensities hereafter are termed as “low” and “high” light treatments. The air temperature was set to a constant 25 °C under both light regimes.

For experimental work, cultures were acclimated for at least 4 weeks to the test conditions with a transfer of each subculture to fresh nutrient medium every 7 days. As a starting inoculum for the growth trials, one healthy colony of a clone was transferred from the 7-day-old subculture into an 80 mm (Ø) crystallizing dish. The vessels contained 100 mL of autoclaved Steinberg medium and were covered with plastic Petri dishes to limit evaporation and contamination. Though the experiments were conducted non-axenically, the axenic stock cultures and autoclaved medium ensured that microbial or algal contamination did not affect plant development during the experimental period. The static experiments were conducted for 7 days, after which growth, morphometric, and photosynthetic traits of the plants were analyzed.

### 2.2. Growth and Morphological Traits

Growth of the cultures was characterized by calculating relative growth rates (RGRs). On the starting (0th) and finishing (7th) days of the experiments, the cultures were photographed perpendicularly by means of a cell phone camera (Samsung Galaxy S22, 12 Mpx, ~29 px mm^−1^ in the applied setup) and a custom-built light box. From the obtained images, frond and colony numbers were counted manually with the “Cell counter” function of ImageJ 1.54d [37].

Relative growth rates were calculated according to OECD [38] as follows:

RGR_frond_ (day^−1^) = (lnFN_7_ − lnFN_0_)/t

where lnFN_0_ and lnFN_7_ are natural logarithms of the respective frond numbers on experimental days 0 and 7, while t is the duration in days between the two counts (i.e., t = 7), respectively.

The proportion of the senescent fronds was determined by counting fronds that showed any sign of yellowing (Scheme 1), and by calculating their percentile ratio relative to the total frond numbers.

Colony size, that is the mean number of fronds comprising one colony were calculated by dividing the number of fronds by the number of colonies in a vessel on the 7th day of the trial, as follows:

colony size (frond colony^−1^) = FN/CN

where FN and CN are the numbers of fronds and colonies in a vessel on the respective experimental day.

The length and width of mature fronds were measured in the same photographs that were used to determine frond and colony numbers on the 7th day of experiments. For that purpose, 5 representative mature fronds that were the largest ones within their colony and were fully emerged from their original parental frond’s meristematic pocket were selected in each vessel. Their length between the basal and apical tips and their width at the widest part were determined in ImageJ [37]. From length and width data, the area of fronds was estimated by assuming an ellipsoid form as follows:

A = a × b × π

where “a” and “b” are the half-length and half-width of the frond, respectively.

Roundness of fronds was characterized by calculating their length-to-width ratio, with a ratio of 1 denoting perfectly circular fronds.

Morphometric data from 5 fronds were then averaged on a vessel basis.

### 2.3. Chlorophyll Fluorescence

#### 2.3.1. Fast Chlorophyll Fluorescence Induction Parameters

Fast chlorophyll fluorescence (ChlF) induction was studied by means of a PAR-Fluorpen FP110/P (Photon Systems Instruments spol. s.r.o., Drásov, Czech Republic) fluorometer after 20 min of dark adaptation. The measurements were performed by using the instrument’s built-in OJIP protocol that applied a ~2 s long saturating light pulse (blue, peak intensity at 470 nm) while recording the fluorescence yield of samples at high frequency (JIP-test) [39,40]. The derived and analyzed parameters from these measurements are detailed in Table 2. From each vessel, 3 subsamples were subjected to the JIP test, and their results were then averaged on a vessel basis.

#### 2.3.2. PAM Fluorometry and Chlorophyll Fluorescence Imaging

ChlF induction kinetics were analyzed by means of PAM fluorometry. A Maxi Imaging-PAM (Waltz GmbH, Effeltrich, Germany) fluorometer was used. The plants were dark-adapted in their vessels for 20 min prior to measurements. After this period, a 10 min long kinetic curve measuring protocol was performed to record both the dark- and light-adapted ChlF parameters [41]. From the basic ChlF parameters, yields of dark- and light-adapted photochemical energy conversion [Fv/Fm and Y(II)], regulated [Y(NPQ)], and non-regulated non-photochemical quenching [Y(NO)] were calculated ([42] Table 2), respectively. In order to mimic their ambient light environment, 81 and 231 μmol m^−2^ s^−1^ nominal actinic light intensities were applied for the low-light and high-light samples, respectively.

Following the measurement of slow ChlF induction kinetics, the instrument’s built-in rapid light curve (RLC) protocol was initiated to record ChlF parameters at 13 incremental actinic light intensities between 0 and 700 μmol m^−2^ s^−1^, maintaining each light intensity for 20 s prior to measurement [41]. Electron transport rate (ETR) at each light intensity was calculated as follows [43]:

ETR (μmol e^−^ m^−2^ s^−1^) = Y(II) × PPFD × 0.5 × 0.84

where Y(II) is the actual photochemical energy conversion rate (see Table 2), PPFD is the applied actinic light intensity, 0.5 represents an assumed stoichiometric distribution of the absorbed excitation energy between the two photosystems, and 0.84 is an estimated empirical light absorbance efficiency of plants, respectively.

The dependency of ETR on PPFD was described by fitting saturation curves in Sigmaplot 14.0 (Systat Software Inc., San Jose, CA, USA) by means of its built-in exponential rise to max function [44]. From the fitted RLC, the maximal light use efficiency (α), the relative maximum of electron transport rate (rETR_max_), and the onset of light saturation (Ek) were derived (Table 2).

For the above measurements, the instrument’s built-in LED light source (blue, 450 nm peak intensity) was applied. The derived parameters are explained in detail in Table 2.

**Table 2:** The studied chlorophyll fluorescence parameters as derived from the fast and slow ChlF induction kinetics and rapid light curves, respectively.

Protocol	Parameter	Physiological Interpretation	Reference
Fast chlorophyll fluorescence kinetics	Sm	Normalized area—an assumed proportional to the number of reductions and oxidations of one Q_A_-molecule during the fast OJIP transient, and therefore related to the number of electron carriers per electron transport chain	[39,45]
	Phi_Pav	Time needed to reach maximal ChlF yield (in ms)	[39,45]
	Pi_Abs	Performance index on absorption basis for energy conservation from photons absorbed by PSII antenna to the reduction of Q_B_	[39,45]
	ABS/RC	Absorption flux per reaction center	[39,45]
	TRo/RC	Maximum trapped exciton flux per active PSII unit; inversely proportional to the number of PSII reaction centers in the sample	[39,45]
	ETo/RC	Electron transport flux per reaction center	[39,45]
	DIo/RC	Dissipated energy flux per reaction center	[39,45]
	Fv/Fm	Maximal (i.e., dark-adapted) photochemical energy conversion rate	[39,45]
Slow chlorophyll fluorescence kinetics	Y(II)	Actual (i.e., adapted to the ambient irradiation) photochemical energy conversion rate	[42]
	Y(NPQ)	Regulated non-photochemical quenching; proportional to the efficiency of photoprotective mechanisms	[42]
	Y(NO)	Non-regulated non-photochemical quenching; proportional to constitutive energy losses in photochemistry	[42]
Rapid light curve	α	Initial slope of the RLC, that is, the maximal light use efficiency; the number of electrons moved through the electron transport chain by 1 absorbed photon (as e^−^ photon^−1^)	[46]
	rETR_max_	Maximal rate of the electron transport under the applied ambient conditions (as μmol e^−^ m^−2^ s^−1^)	[46]
	Ek	Onset of light saturation in the course of RLC (as μmol m^−2^ s^−1^)	[46]

#### 2.3.3. Chlorophyll Fluorescence Image Analysis

**Figure 1:**
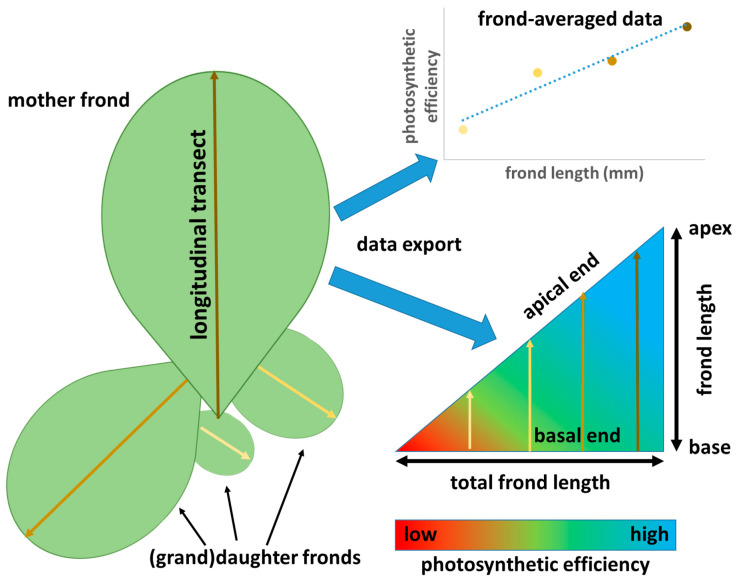
Extraction and processing of spatially resolved chlorophyll fluorescence data sampled along the longitudinal axis of mother, daughter, and granddaughter fronds (2nd generation daughter frond), respectively. Pixel-level data were used to both derive frond-level averages for correlation analyses (top-right plot) and to analyze within-frond patterns in photosynthetic efficiency (bottom-right plot), respectively. Note: the longitudinal transect of each frond in the colony has a distinctive shade that is also used in the plots on the right side.

The images (640 × 480 pixels, 4.6 pixel mm^−1^ resolution in the applied setup) from the end of the slow ChlF induction kinetics were used to analyze frond-level photosynthetic traits of the cultures. The methodology followed that reported by Oláh et al. [25]. In brief, Y(II), Y(NPQ), and Y(NO) were measured and extracted along longitudinal transects of the fronds that were drawn from the basal towards the apical tip (Figure 1). The obtained pixel-by-pixel data (spatial position as distance from the frond base and value of the given parameter), as well as frond-averaged data (total frond length, average value of the given parameter), were compiled in a database and processed further. For measurements, 7–8 colonies corresponding to a total of at least 30 fronds with various sizes were randomly selected and analyzed in each vessel.

### 2.4. Biomass Sampling

After photographing the cultures and measuring chlorophyll fluorescence induction parameters, 10–60 mg of fresh biomass was sampled from each vessel for photosynthetic pigment content assays. The rest of the biomass in every vessel was used for determining dry matter contents. For that, the plant surface area for the corresponding biomass samples was determined in ImageJ [37]. The plants were then gently blotted with paper towels, and the fresh weight of the samples was measured with 0.1 mg accuracy. The samples were then dried for 72 h at 65 °C before determining their dry mass (DM). Frond mass-to-area ratio (FMA) was calculated using the measured plant areas and dry masses as follows:

FMA (mg cm^−2^) = DM/Area

### 2.5. Measurement of Photosynthetic Pigment Contents

The sampled colonies were flash-frozen in liquid N_2_ and stored at −80 °C until further processing. After their homogenization in liquid N_2_, photosynthetic pigments were extracted overnight in 2 mL chilled acetone:water:NH_4_OH (80:20:0.1 *V*/*V*). The concentrations of chlorophyll-a (Chl-a), chlorophyll-b (Chl-b), and carotenoids (Car) were spectrophotometrically determined according to Wellburn [47] by using absorbances of the aliquots at 470.0, 646.8, 663.2, and 750.0 nm and expressing the concentrations on a fresh mass basis.

Carotenoid composition was analyzed in 20 µL of the same aliquots by means of reversed-phase HPLC (Jasco, Tokyo, Japan), according to Láposi et al. [48] and Oláh et al. [49]. The concentration of carotenoid compounds was expressed on a chlorophyll basis (as mmol Car mol^−1^ Chl-a + b).

### 2.6. Data Analysis and Statistics

The experiments with each clone and light intensity were performed in triplicate vessels and were repeated twice (*n* = 6). The respective values for growth, morphometric, and ChlF parameters were pooled from the two experiments, resulting in an *n* = 6 sample size for statistical analyses. The photosynthetic pigment composition was analyzed in 2 samples per experiment, and the results were pooled afterwards, resulting in an *n* = 4 sample size.

Possible effects of clonal origin and light environment on plant responses, and any possible interaction, were tested by means of two-way analysis of variance (ANOVA). Prior to that, homogeneity of variances and normality of distribution were checked with Levene’s and Shapiro–Wilk tests, and, in case of violation of those preconditions, data distribution was improved by log-transformation. In addition to two-way ANOVA, the significance of the two factors and their interaction was also tested by their omega-squared (ω^2^) effect size. When the two-way ANOVA indicated significant interaction of clonal origin with light environment, low- and high-light-grown cultures of the studied clones were compared separately by means of one-way ANOVA. When the two factors did not interact significantly, but two-way ANOVA indicated a significant effect of clonal origin, overall means of low- and high-light-grown cultures of the respective clones were compared by one-way ANOVA, or, in the case of photosynthetic pigment concentrations, the Kruskal–Wallis test. When clonal differences were tested in post hoc pairwise comparisons (Tukey test or Mann–Whitney test), Bonferroni correction was also applied. In case the two-way ANOVA indicated a significant effect of light, variances and means of the low- and high-light-grown cultures of each clone were compared by two-sample F- and t-tests, respectively.

Possible ontogeny-dependent trends in ChlF parameters were tested by fitting linear regression models, using frond-averaged data and frond lengths.

For the above analyses, Past 4.0 [50] statistical software was used, and *p* < 0.05 was considered as the threshold for statistical significance.

Within-frond patterns in ChlF data were visualized by interpolating and plotting pixel-level data in RStudio 2023.06.01 Build 524 [51], using the ‘akima’ [52] and ‘fields’ [53] packages. For that, the respective pixel values were arranged along the longitudinal transects of fronds starting from the base towards the tip (y-axis), and the transects were arranged in an incrementing order of size (x-axis) (Figure 1). During the interpolations, medians of overlapping pixels were used. To ensure sufficient spatial resolution and reduce possible biases from outlier data, the minimal frond length was set to 6 pixels (i.e., >1.0 mm), and the largest 1–2 size classes of fronds in every clone × treatment combination were excluded due to the low sample sizes.

## 3. Results and Discussion

### 3.1. Growth and Morphology

**Figure 2:**
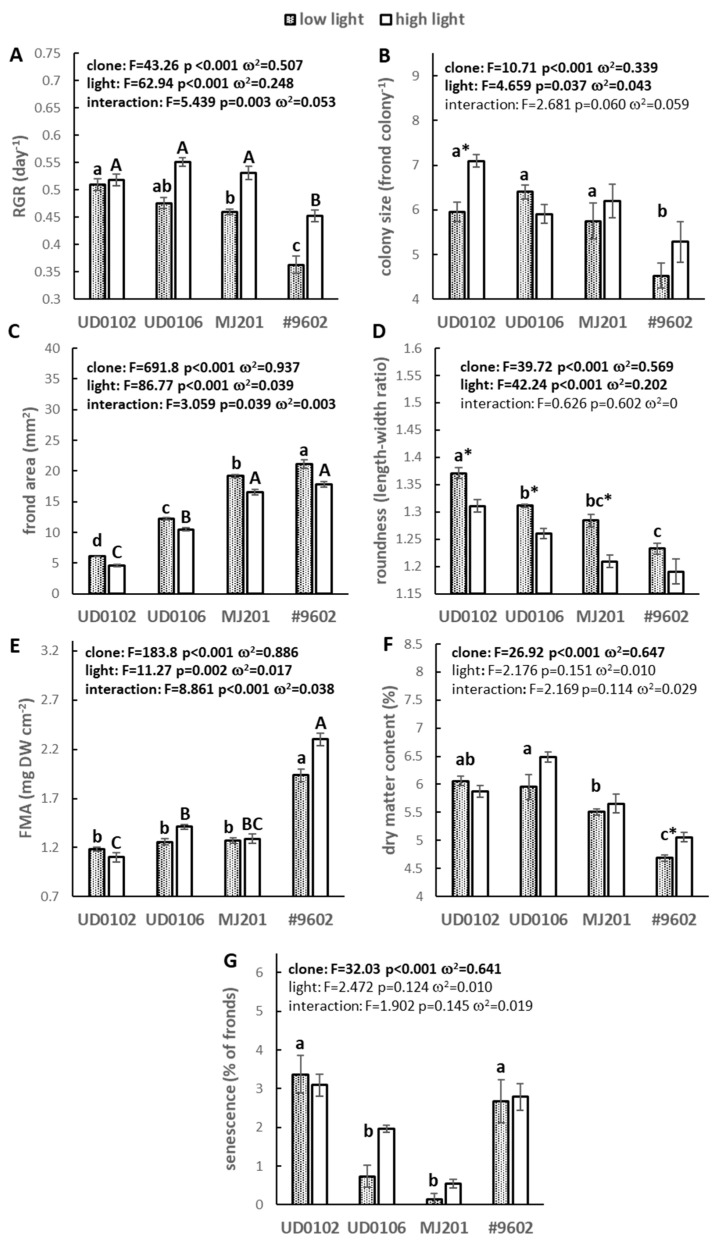
Frond number-based relative growth rates—RGR (**A**), morphological traits like colony size (**B**), frond area (**C**), and roundness (**D**), frond mass-to-area ratio (FMA, (**E**)), dry matter content (**F**), and occurrence of senescence (**G**), in the studied *L. gibba* clones under low- (shaded columns) and high-light intensities (open columns), respectively. Means ± SE of *n* = 6 samples; “clone”, “light”, and “interaction” indicate the effects of genotype, light intensity, and their interaction according to two-way ANOVA (significant effects in bold). Different lower and upper cases denote significantly (*p* < 0.05) different means of clones within the respective light treatment when two-way ANOVA indicated significant interaction of the two factors. When the interaction between clone and light was not significant, different lower cases denote significant differences of overall (both low- and high-light) means of the clones according to the one-way ANOVA and post hoc Tukey test. Asterisks indicate significantly different means of low- and high-light-grown cultures of the respective clone when there was no significant interaction between clone and light.

The studied *L. gibba* clones displayed rapid growth under the applied experimental conditions. Even the slowest-growing cultures (clone #9602 under low-light) doubled their frond numbers in <2 days (i.e., RGR = 0.36 day^−1^), while the fastest-growing combination (UD0106 under high-light) needed less than 1.3 days (RGR = 0.55 day^−1^) as a doubling time (Figure 2A). In general, higher light intensity promoted growth, but not in all clones: low- and high-light-grown cultures of UD0102 did not differ from each other in terms of RGR (*t*-test *p* = 0.59, Figure 2A). It was reported earlier that duckweeds can flexibly acclimate to various light environments [17,54], but their growth rates tend to saturate at relatively low daily light integrals [18]. Our results showed that the lower applied light level in this study (100 μmol m^−2^ s^−1^) was still below that saturation point, and growth rates improved by >10% as an average across all clones in response to higher light intensity (i.e., 243 μmol m^−2^ s^−1^). Yet, the existence of clonal differences in the light saturation point can be hypothesized as demonstrated by the differential response of clone UD0102 [55]. When exceeding light saturation, duckweeds had been reported to maintain stable RGR with no signs of photoinhibition up to very-high-light intensities [18]. The excess energy, therefore, needs to be utilized in alternative ways such as enhanced respiration and accumulation of starch [17,18], avoided by reduced chlorophyll content (i.e., lower light absorption), or released via photoprotective processes [21]. Interestingly, in high-light-grown cultures of UD0102, neither the dry mass content and FMA as indirect proxies for starch content nor pigment composition (discussed in detail in Section 3.4) were indicative of how excess light was safely managed in this clone. This question is, thus, yet to be resolved in future work.

In general, every measured frond morphological trait showed strong genotype dependence, as supported by the larger effect sizes of the clonal origin compared to those of light (Figure 2B–D). The two Hungarian clones (UD0102 and UD0106) had smaller and more elongated fronds, somewhat larger colonies, and higher dry matter content than MJ201 and #9602, respectively. Clone #9602 also sticks out from the other clones with its considerably higher FMA, which suggests thicker fronds, possibly as a result of tetraploidy [28]. In a previous study, the tetraploid #9602 clone displayed larger and more roundish fronds but lower dry matter content compared to its ancestor clone #7796 [31], suggesting a smaller FMA. Triploid *L.* × *mediterranea* clones, on the other hand, were reported to have similar frond size and length-to-width ratio as the parental species *L. gibba* [29]. The morphological differences of MJ201 compared to the Hungarian clones, therefore, could rather be attributed to natural variance in these traits than an outcome of its triploid genome.

The light regime had significant effects on colony and frond sizes, frond shape, and FMA, though their effect sizes proved to be smaller than those of genotype. As an overall trend, the fronds became smaller, more round, denser, and thicker (increasing dry matter content and FMA) and also stayed longer connected together (increasing colony size) under the higher light intensity (Figure 2B–F). These results all point towards the strong effect of light environment on frond ontogenesis, similar to previous observations on gibbosity and length-to-width ratio as a function of light [55]. Of the above traits, however, interaction of genotype and light was only confirmed statistically in the case of frond area and FMA, and the effect sizes for even those parameters stayed small (Figure 2C,E).

Interestingly, senescence proved to strongly depend on the genotype, but not on the light environment. Recent findings with *L. minor* suggest that light intensity—or DLI—has an influence on frond lifespan due to changing the caloric intake [56]. Yet, in our experiments, we found no such effect, perhaps because light treatments were not sufficiently contrasting. Our results, on the other hand, indicate that polyploidization did not necessarily imply shorter frond lifespans: #9602 had a somewhat higher senescence rate than UD0106 but did not significantly differ from clone UD0102 (Figure 2G).

### 3.2. Fast Chlorophyll Fluorescence Induction

**Figure 3:**
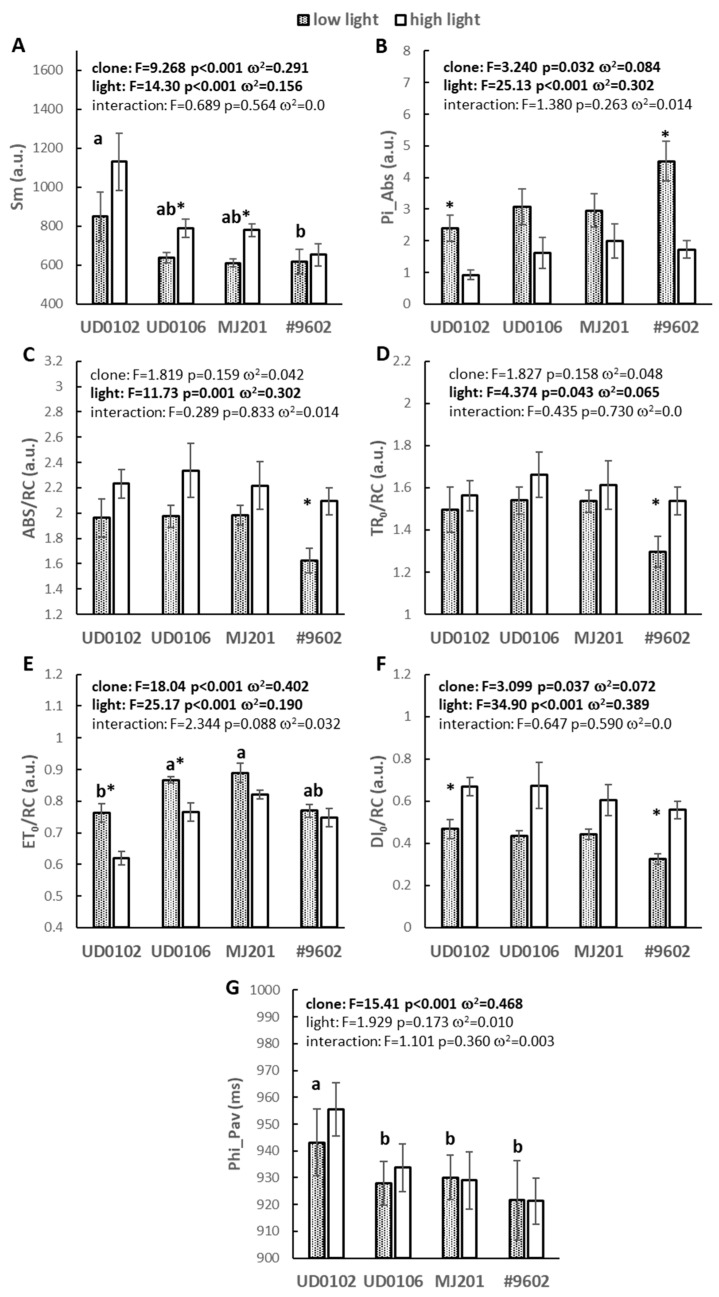
The number of electron carriers per reaction center (Sm, (**A**)), performance index on absorption basis (Pi_Abs, (**B**)), absorption (ABS/RC, (**C**)), maximum trapped exciton flux (TR_o_/Rc, (**D**)), electron transport flux (ET_o_/RC, (**E**)), and dissipation of energy fluxes per reaction center (DI_o_/RC, (**F**)), and time needed to reach maximal fluorescence (Phi_Pav, (**G**)) in the studied *L. gibba* clones under low- (shaded columns) and high-light intensities (open columns), respectively. The parameters were defined in Table 2. Means ± SE of *n* = 6 samples; “clone”, “light”, and “interaction” indicate the effects of genotype, light intensity, and their interaction according to two-way ANOVA (significant effects in bold). Different lower cases denote significantly (*p* < 0.05) different overall means of the clones (merged low- and high-light cultures) according to the one-way ANOVA and post hoc Tukey test due to the non-significant interaction between clone and light. Asterisks indicate significantly different means of low- and high-light-grown cultures of the respective clone.

Fast ChlF induction parameters, in general, indicated both strong genotypic and environmental (light) influences on light absorption and primary energy conversion (Figure 3). The time needed to reach maximal fluorescence (Phi_Pav, Figure 3G) was the only parameter that was not significantly affected by light intensity. The rest of the parameters reflecting absorption and trapping processes proved all to be responsive to the light treatment with large omega-squared effect sizes, with the exception of the number of reaction centers in the sample (TR_o_/Rc, ω^2^ = 0.065). The observed trends are in agreement with those by Lepeduš et al. [57], who studied responses of *L. minor* to more diverging light environments (50 vs. 500 μmol m^−2^ s^−1^) and reported very similar patterns. Genotype, in contrast, had an effect on all studied parameters except ABS/RC (absorption flux on reaction center basis) and TR_o_/RC. It should also be noted that the significance of the genotype effect was marginal for both Pi_Abs (performance index on absorption basis) and DI_o_/RC (dissipated energy flux per reaction center), which could not be confirmed by the Bonferroni-corrected post hoc pairwise comparisons, and their omega-squared effect sizes stayed relatively small. We found no previous literature that compared OJIP parameters in different duckweed species or clones, but results with rice (*Oryza sativa*) and barley (*Hordeum vulgare*) cultivars confirm that genotype-dependence is indeed a factor in such assays [58,59]. Despite the influence of both light and genotype on most parameters, however, no significant interaction of the two was found. Different clones responded in consonant ways to increased light intensity irrespective of their cytotype, though the changes were not always significant when low- and high-light-grown cultures of the respective clones were compared (Figure 3). The number of reaction centers (TR_o_/Rc), the number of electron carriers per reaction center (Sm), and both the absorption (ABS/RC) and dissipation of energy fluxes per reaction center (DI_o_/RC) increased in general. ABS/RC is considered to be proportional to the average antenna size. Its increase under higher light intensity, therefore, seemingly contradicts the expected response but can be explained by a possible overload in PSII reaction centers [57,60]. The electron transport flux per reaction center (ET_o_/RC) was down-regulated in response to elevated light. Similarly, performance index (PI_Abs) decreased due to exposure to excess light [60].

### 3.3. PAM Fluorometry

**Figure 4:**
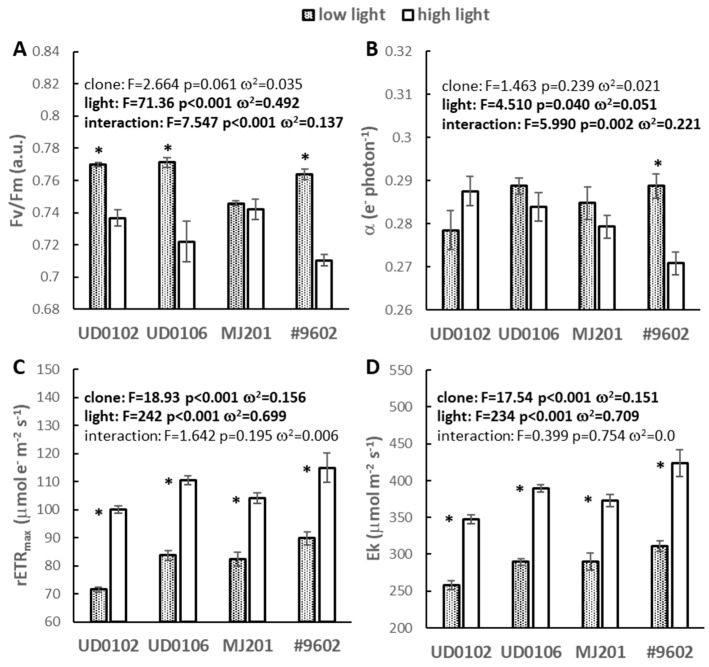
Potential photochemical efficiency (Fv/Fm, (**A**)), maximal light use efficiency (α, (**B**)), relative maximum of electron transport rate (rETR_max_, (**C**)), and onset of light saturation during the performed rapid light curves (Ek, (**D**)) in the studied *L. gibba* clones under low- (shaded columns) and high-light intensities (open columns), respectively. Means ± SE of *n* = 6 samples; “clone”, “light”, and “interaction” indicate the effects of genotype, light intensity, and their interaction according to two-way ANOVA (significant effects in bold). Different lower and upper cases denote significantly (*p* < 0.05) different means across clones under low-light (lower cases) and high-light (upper cases) conditions according to one-way ANOVA and post hoc Tukey tests when the interaction between clone and light was significant. When only lower cases are used, overall means of the clones (merged low- and high-light cultures) were compared due to non-significant interaction between clone and light. Similarly, asterisks indicate significantly different means of low- and high-light-grown cultures of the respective clone when light had a significant effect but no interaction with the clone.

No genotypic effect was found in the case of maximal photochemical energy conversion rate (Fv/Fm) and maximal light use efficiency (α), but both parameters reflected light regime-dependent patterns and had higher values in low-light plants in photosynthetic energy conversion (Figure 4A,B). In the case of α, higher values can be attributed to an improved light utilization under weaker irradiation [61], but the effect size of this response stayed small. Lowered Fv/Fm in high-light plants may reflect photosynthetic down-regulation or photoinhibition due to increased energy load on reaction centers [62]. It should also be noted that not all clones responded in the same way: low- and high-light-grown MJ201 showed no significant difference in Fv/Fm, while the light response of α proved only to be significant in clone #9602 (Figure 4A,B). In contrast, the maximal electron transport rate (rETR_max_) and the onset of light saturation (Ek) indicated better performance of high-light-adapted plants under strong irradiation (Figure 4C,D), just as it could be presumed based on the previous literature data [63]. These parameters reflected strong genotype dependency, but with lower effect sizes than those of light, and no significant interaction was found between the two factors.

### 3.4. Photosynthetic Pigment Composition

The fresh mass-based photosynthetic pigment concentrations are presented in Table 3. According to the performed two-way ANOVA, Chl a + b content was both clone- and light regime-dependent, with clone being the stronger factor based on its effect size. The two factors, however, did not significantly interact. Similarly, according to their effect sizes, clonal origin proved to be the stronger driver when Chl a and Chl b concentrations were analyzed separately (Table 3). In the case of Chl a, the two factors showed significant interaction, but its effect size was rather small. The fresh-mass-based total Car content, on the other hand, showed only genotype-related differences; the light environment did not have a significant effect, and neither did the two factors interact (Table 3). Based on these results, in agreement with the literature, diploid UD0102 and UD0106 had higher pigment contents as compared to MJ201 and #9602, respectively [28,31]. Considering the effects of light, in general, low-light-grown cultures contained more chlorophyll on a fresh mass basis, though the differences did not prove to be significant in every clone (Table 3). This light response can be regarded as typical, and duckweeds’ flexible acclimation to contrasting light environments—most probably due to their evolutionary history—is well known [21]. The only exception was clone #9602, which had comparable Chl a + b content irrespective of the light environment (Table 3).

**Figure 5:**
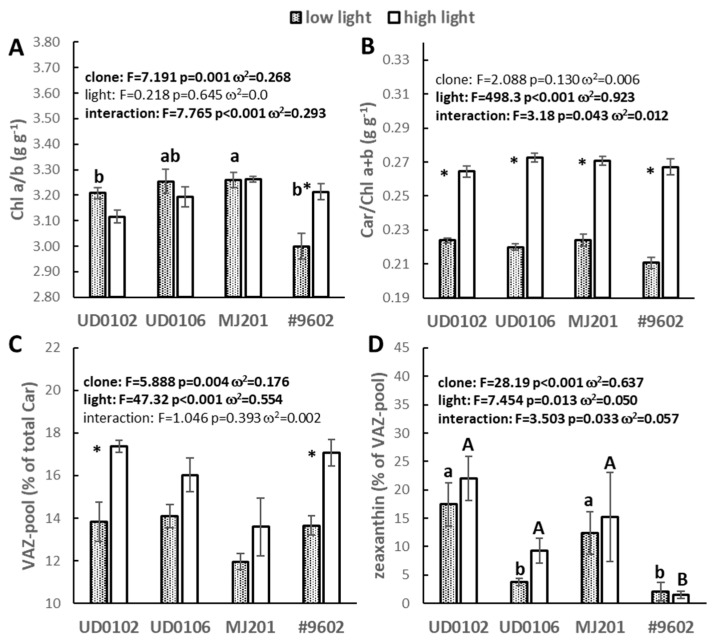
Photosynthetic pigment composition of the studied *L. gibba* clones under low- (shaded columns) and high-light intensities (open columns). Relative amounts of different pigment components are expressed as ratios of chlorophyll a to b (Chl a/b, (**A**)) and total carotenoids to total chlorophyll (Car/Chl a + b, (**B**)), the relative share of the VAZ-pool (Violaxanthin + Antheraxanthin + Zeaxanthin) within total carotenoids (**C**), and the share of Zeaxanthin within the VAZ-pool (**D**). Means ± SE of *n* = 4 samples; “clone”, “light”, and “interaction” indicate the effects of genotype, light intensity, and their interaction according to two-way ANOVA (significant effects in bold). Different lower and upper cases denote significantly (*p* < 0.05) different medians across clones under low-light (lower cases) and high-light (upper cases) conditions according to Kruskal–Wallis and post hoc Mann–Whitney tests, when the interaction between clone and light was significant. When only lower cases are used, overall medians of the clones (merged low- and high-light cultures) were compared by Kruskal–Wallis and post hoc Mann–Whitney tests due to non-significant interaction between clone and light. Similarly, asterisks indicate significantly different means of low- and high-light-grown cultures of the respective clone when light had a significant effect but did not interact with the clone.

Cultures of #9602 also stood out from the rest of the clones in terms of the Chl a/b ratio: this was the only clone that showed significant change in response to the light treatment (Figure 5A). These results show that tetraploidization might have had an effect on the pigment composition of plants, consistent with the literature [31]. In contrast with Chl a/b ratios, the relative abundance of carotenoids compared to chlorophylls (Car/Chl a + b), the share of the Violaxanthin + Antheraxanthin + Zeaxanthin-pool size within total carotenoids (VAZ/Car), and the share of Zeaxanthin within the VAZ-pool (Z/VAZ) indicated an increased demand for photoprotection in high-light plants of all clones (Figure 5B–D, [20]). Two-way ANOVA revealed that VAZ/Car and Z/VAZ were dependent on the light regime as well as genotype, though the interaction between the two factors was only found to be significant in the case of the latter parameter, and the effect size for this interaction was small. It should also be noted that, even though two-way ANOVA indicated significant influence of clone and light on the VAZ-pool size, pairwise comparisons could only confirm this effect for clones UD0102 and #9602 in the case of light, and no significant difference was found across clones when the Bonferroni-corrected post hoc Mann–Whitney test was performed (Figure 5C). In clones UD0102, UD0106, and MJ201, Zeaxanthin comprised a somewhat larger portion of the VAZ-pool as compared to #9602 (Figure 5D). Smith et al. [54] found that duckweed light acclimation can be shaped by microevolutionary drivers that might have supported adaptation of different clones to rather shaded or sunlit habitats. Our results also point to the fact that, in addition to high phenotypic plasticity, duckweed clones might follow, in part, individual strategies in light acclimation.

### 3.5. Ontogenetic Acclimation of Fronds to Ambient Light

**Figure 6:**
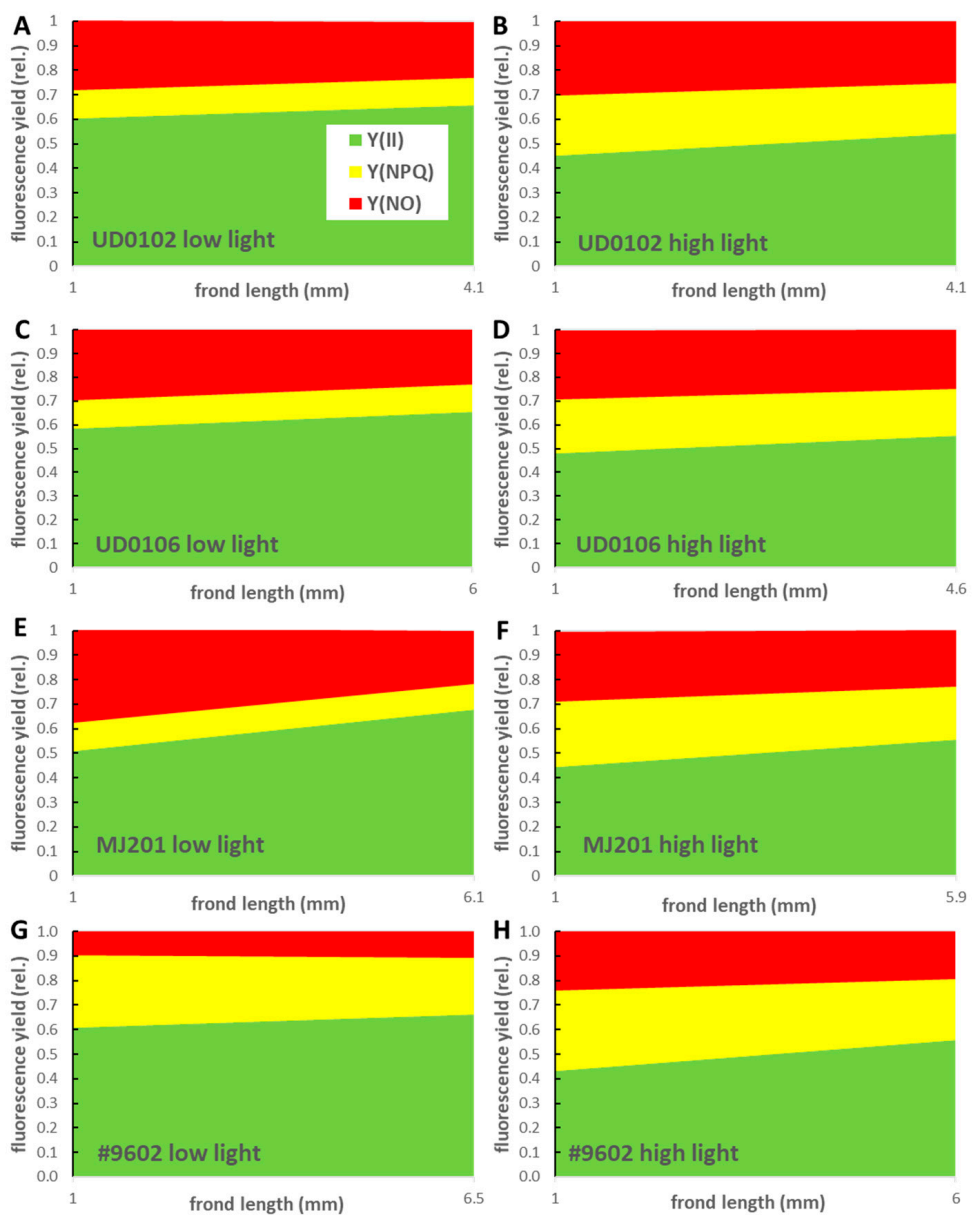
Patterns in the allocation of absorbed excitation energy in *L. gibba* fronds during their maturation. Y(II) (green) denotes photochemical quantum yield (i.e., photosynthesis), while Y(NPQ) (yellow) and Y(NO) (red) denote proportions of regulated and non-regulated photochemical quenching, respectively, as functions of frond length (x-axis). The four *L. gibba* clones UD0102 (**A**,**B**), UD0106 (**C**,**D**), MJ201 (**E**,**F**) and #9602 (**G**,**H**) were cultivated under low- (**A**,**C**,**E**,**G**) and high-light treatments (**B**,**D**,**F**,**H**), and the trends in quenching processes were described by linear regression models (see supplementary Figure S3 and Table S2).

Photochemical efficiency—Y(II)—was, in general, higher in low-light-grown fronds of either clone as compared to the high-light-grown ones (one-way ANCOVA *p* < 0.0001 for each clone). Based on the fitted linear regression models, Y(II) increased in all clones in the course of frond expansion (Figure 6; the model fittings are summarized in supplementary Figure S3 and Table S2). This pattern most likely reflects parallel maturation and acclimation of the photosynthetic machinery in developing duckweed fronds and results in gradually improving photochemical efficiency [23,25]. Under high-light conditions, the pattern was very similar across all clones. It can be observed that 1 mm long fronds displayed Y(II) in the range of 0.43–0.48, which increased to 0.54–0.56 in the mature-sized ones. Under low-light conditions, efficiency in 1 mm long fronds was more variable, ranging from 0.51 (MJ201) to 0.61 (#9602). Despite those initial differences, Y(II) in mature low-light fronds was rather similar, ranging between 0.66 and 0.68. One would expect that lower Y(II) in young fronds was coupled with a higher demand for photoprotection [64]. Interestingly, in most clones, building up of Y(II) did not happen at the expense of Y(NPQ), but rather that of Y(NO) (Figure 6). In low-light-grown fronds, Y(NPQ) remained nearly constant (<0.01-unit change) throughout frond expansion and maturation. A possible explanation can be that the xanthophyll-cycle-related Y(NPQ) stayed stable due to the applied continuous irradiation, which allowed fronds to maintain steady-state photosynthesis all day round with a limited need to dynamically regulate photoprotection. Alternatively, improved photoprotection by such other mechanisms as chlorophyll fluorescence can also be hypothesized in the early stage of frond ontogeny. Peršić et al. [26] found that energy dissipation (DI_0_/RC) during the rapid phase of chlorophyll fluorescence induction gradually decreased in maturing *Spirodela polyrhiza* fronds. Either way, further research is needed to better understand photoprotection of fronds developing under various ambient conditions, as it seems to be fundamental in successful acclimation of these plants. The only exception to this pattern was clone #9602. The decrease in Y(NPQ) in this clone exceeded >0.06 unit during the maturation process, in contrast with a <0.01 unit change in Y(NO) (Figure 6G). In high-light-grown fronds, on the other hand, a simultaneous decrease in both Y(NPQ) and Y(NO) was observed. In most clones, the decrease in Y(NO) exceeded that of Y(NPQ) (Figure 6B,D,F). The exception was again clone #9602, in which Y(NPQ) shrunk by 0.07 unit, while Y(NO) decreased by only 0.04 (Figure 6H). Our results, thus, point to a distinct light acclimation strategy of clone #9602, which strongly relied on photoprotective mechanisms at the early stage of frond development and gradually tuned Y(NPQ) down with improving photosynthetic efficiency. Interestingly, higher Y(NPQ) in young fronds of clone #9602 was not reflected by an enhanced VAZ-pool or higher proportion of Zeaxanthin in the biomass as one would assume [20]. This contradiction might be explained by the low relative growth rate of this clone amongst the tested ones. A lower RGR means a lower proportion of young fronds in the biomass. Additionally, this clone had the highest frond mass-to-area (FMA), which further decreased the relative share of expanding fronds within the pooled biomass for pigment assays. This way, a higher proportion of mature fronds with a lower demand for Y(NPQ) might have obscured the more dynamic VAZ-pool in younger fronds in this clone.

**Figure 7:**
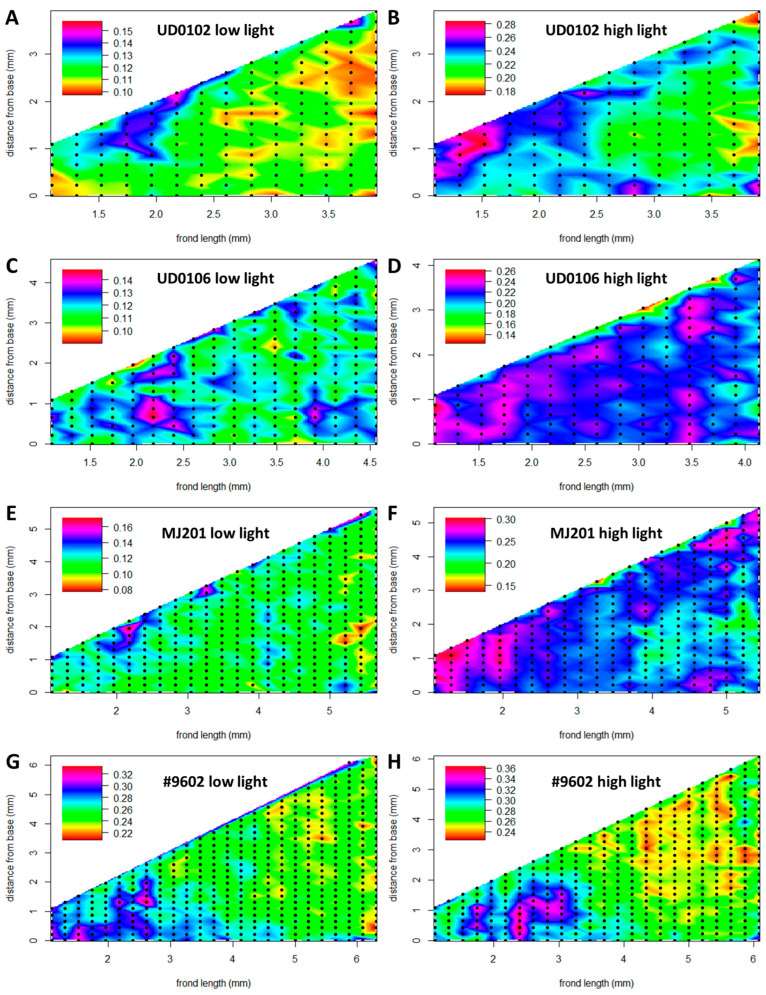
Within-frond patterns in actual photochemical efficiency [Y(II)] during ontogenesis of the studied four *L. gibba* clones UD0102 (**A**,**B**), UD0106 (**C**,**D**), MJ201 (**E**,**F**) and #9602 (**G**,**H**), under low- (**A**,**C**,**E**,**G**) and high-light treatments (**B**,**D**,**F**,**H**), respectively. The interpolation plots are composed of pixel-by-pixel Y(II) values along longitudinal transects in fronds with different sizes. Frond size classes are arranged on the x-axis, while within-frond position measured from the basal tip is plotted on the y-axis. Black dots denote positions of the actually measured pixels along the transects. Note: color gradients are set individually for each plot.

When Y(II) was plotted as a combined function of frond size and location within the frond, very similar patterns emerged irrespective of genotype and light environment (Figure 7). Photosynthetic efficiency stayed relatively low until reaching ~50–60% of the mature frond length. From that point, distinct zones appeared within the frond: the middle and apical parts displayed higher Y(II) in contrast with a lower performance of the basal zone (Figure 7). The basipetal gradient in Y(II) was more pronounced in young, low-light-grown fronds. When reaching ~2.5–3 mm in length, the zone of higher photosynthetic performance appeared at the apical (ontogenetically more mature) part of the fronds and gradually spread towards the middle and basal sections in parallel with further frond elongation (Figure 7). In high-light-acclimated fronds, this transition was much sharper: the higher-performing zone appeared almost simultaneously along the entire frond axis. The only exception in this sense was clone UD0102, in which the high-light-grown fronds showed the same basipetal pattern as the low-light-grown ones (Figure 7B).

The enigmatic body plan of duckweeds has been puzzling botanists for centuries [11,65,66]; fronds were either interpreted as the derivative of a leaf, a shoot, or the combination of those two. Landolt [11] hypothesized that the basal frond part was composed of a very short shoot that ended at the node, while the distal part was the remnant of either a petiole or a leaf branching off of this shoot. Similarly, recent findings by Ware et al. [67] supported this composite structure of duckweed frond. Whether petiole or leaf, the distal frond section showed very similar functional separation from the basal part in our previous study when photosynthetic activity was mapped along longitudinal transects of *S. polyrhiza*, *Landoltia punctata*, and *L. minor* fronds, respectively [25]. The appearance and stabilization of the lower-performing basal part in the interpolated maps, in this regard, may mark full protrusion of the daughter frond from the mother frond’s pocket.

Basipetal patterns in Y(NPQ) of fronds did not show similar trends as Y(II) (Figure S4). Fronds in high-light cultures displayed somewhat higher Y(NPQ) at their early developmental stage that gradually decreased as the fronds expanded and reached their mature size. Increase in Y(II), however, was not due to decreases in Y(NPQ) but rather due to decreases in Y(NO), with the exception of clone #9602. Therefore, the spatial patterns along ontogenesis were less pronounced in this parameter.

## 4. Conclusions

Our results with a limited set of duckweed clones highlighted that even intraspecific clones of the same species and interspecific hybrids of closely related species can follow partially different strategies in acclimation to ambient conditions. The differences may emerge from different ploidy levels, hybridization, and adaptation to previous habitat conditions, as well as other factors yet to be explored in detail. In response to light, this acclimation involved various morphological/anatomic, physiological, and biochemical adjustments and happened in a very short time window at the early life stage when young, still-developing fronds can flexibly achieve an optimized phenotype. This rapid acclimation explains the worldwide success of duckweeds in colonizing very contrasting habitats and also underlines the importance of understanding duckweed responses at the frond level, where the actual acclimation takes place.

## Figures and Tables

**Table 1:** Details of the used *L. gibba* clones in this study.

Clone	Origin	Note
UD0102	Poroszló, Hungary (47.646, 20.664)	
UD0106	Hajdúbagos, Hungary (47.386, 21.654)	
MJ201	Mt. Lucas, Ireland (53.275, −7.209)	triploid *Lemna* × *mediterranea*
#9602	mutant of 7796 (G3, Sicily, Italy), originally from the Khurana lab (New Delhi, India)	spontaneous tetraploid [31]

**Table 3:** Fresh mass-based concentrations of the main photosynthetic pigment groups in the studied *L. gibba* clones under low- (“low”) and high-light intensities (“high”). Means ± SE of *n* = 4 samples; “clone”, “light”, and “interaction” indicate the effects of genotype, light intensity, and their interaction according to two-way ANOVA (significant effects in bold), while omega-squared (ω^2^) denotes effect size. Different lower and upper cases denote significantly (*p* < 0.05) different medians across clones under low-light (lower cases) and high-light (upper cases) conditions according to Kruskal–Wallis and post hoc Mann–Whitney tests, when the interaction between clone and light was significant. When only lower cases are used, overall medians of the clones (merged low- and high-light cultures) were compared by Kruskal–Wallis and post hoc Mann–Whitney tests due to non-significant interaction between clone and light. Similarly, asterisks indicate significantly different means of low- and high-light-grown cultures of the respective clone when light had a significant effect.

	Chlorophyll a (mg g^−1^ FW)			Chlorophyll b (mg g^−1^ FW)			Chlorophyll a + b (mg g^−1^ FW)			Total Carotenoids (mg g^−1^ FW)		
UD0102 low	0.723 ± 0.015 a			0.225 ± 0.005 a *			0.949 ± 0.020 a *			0.212 ± 0.003 a		
UD0102 high	0.588 ± 0.029 A			0.188 ± 0.008			0.776 ± 0.037			0.205 ± 0.009		
UD0106 low	0.726 ± 0.030 a			0.223 ± 0.007 a *			0.949 ± 0.037 a *			0.209 ± 0.010 a		
UD0106 high	0.552 ± 0.036 AB			0.173 ± 0.010			0.725 ± 0.047			0.198 ± 0.014		
MJ201 low	0.555 ± 0.006 b			0.170 ± 0.003 b *			0.725 ± 0.009 b *			0.162 ± 0.003 b		
MJ201 high	0.423 ± 0.020 BC			0.130 ± 0.006			0.552 ± 0.026			0.149 ± 0.007		
#9602 low	0.365 ± 0.019 c			0.122 ± 0.005 c			0.486 ± 0.024 c			0.102 ± 0.004 c		
#9602 high	0.349 ± 0.025 C			0.108 ± 0.007			0.457 ± 0.032			0.122 ± 0.010		
two-way ANOVA												
	F	*p*	ω^2^	F	*p*	ω^2^	F	*p*	ω^2^	F	*p*	ω^2^
clone	**65.99**	**<0.001**	**0.706**	**77.96**	**<0.001**	**0.729**	**68.35**	**<0.001**	**0.748**	**64.06**	**<0.001**	**0.845**
light	**40.28**	**<0.001**	**0.142**	**47.93**	**<0.001**	**0.148**	**33.38**	**<0.001**	**0.120**	0.011	0.918	0.0
interaction	**4.012**	**0.020**	**0.033**	2.75	0.066	0.017	2.114	0.126	0.012	**2.548**	**0.081**	**0.021**

Significant (*p* < 0.05) effects of clone, light, and/or their interaction according to two-way ANOVA are highlighted with bold.

## Data Availability

The datasets used in the present study are available from the corresponding author on reasonable request.

## Supplementary Materials

The following supporting information can be downloaded at: https://www.mdpi.com/article/10.3390/plants14182840/s1, Figure S1: Relative genome size measurements of the four *L. gibba* clones studied; Figure S2: Spectral composition of the light environment used in the “low” (100 μmol m^−2^ s^−1^) and “high” light treatments (243 μmol m^−2^ s^−1^), respectively; Figure S3: Allocation of absorbed excitation energy in *L. gibba* fronds during their maturation; Figure S4: Within-frond patterns in the yield of regulated non-photochemical quenching [Y(NPQ)] during ontogenesis of the studied four *L. gibba* clones; Table S1: Calculated relative genome sizes of the studied *L. gibba* clones [68]; Table S2: Parameters of the fitted linear regression models describing trends in photochemical and non-photochemical quenching processes as a function of frond length.

## Abbreviations

The following abbreviations are used in this manuscript:

α	maximal light use efficiency
ABS/RC	absorption flux per reaction center
Car	carotenoids
Chl-a	chlorophyll-a
Chl-b	chlorophyll-b
ChlF	chlorophyll fluorescence
DIo/RC	dissipated energy flux per reaction center
DLI	daily light integral
Ek	onset of light saturation in the course of RLC
ETo/RC	electron transport flux per reaction center
FMA	frond mass-to-area
Fv/Fm	maximal (i.e., dark-adapted) photochemical energy conversion rate
PAM	pulse amplitude modulated fluorometry
Phi_Pav	time needed to reach maximal ChlF yield
Pi_Abs	performance index for energy conservation
PPFD	photosynthetic photon flux density
rETRmax	maximal electron transport rate during an RLC
RGR	relative growth rate
RLC	rapid light curve
Sm	normalized area above the fast chlorophyll fluorescence induction curve
TRo/RC	maximum trapped exciton flux per active PSII unit
VAZ/Car	relative proportion of the VAZ-pool within the total carotenoids
VAZ-pool	the summarized amount of Violaxanthin, Antheraxanthin, and Zeaxanthin
Y(II)	actual (i.e., light-adapted) photochemical energy conversion rate
Y(NO)	non-regulated non-photochemical quenching
Y(NPQ)	regulated non-photochemical quenching
Z/VAZ	relative proportion of Zeaxanthin within the VAZ-pool
