# Supplementary material for: Lemna gibba Clones Show Differences in Phenotypic Responses to the Light Environment
Source: Plants (Basel). 2025 Sep 11;14(18):2840. doi: 10.3390/plants14182840 (PMC12473672; doi:10.3390/plants14182840)
Supplement: Supplementary file 1 [file plants-14-02840-s001.zip › plants-3820953-supplementary.pdf]

Supplementary materials

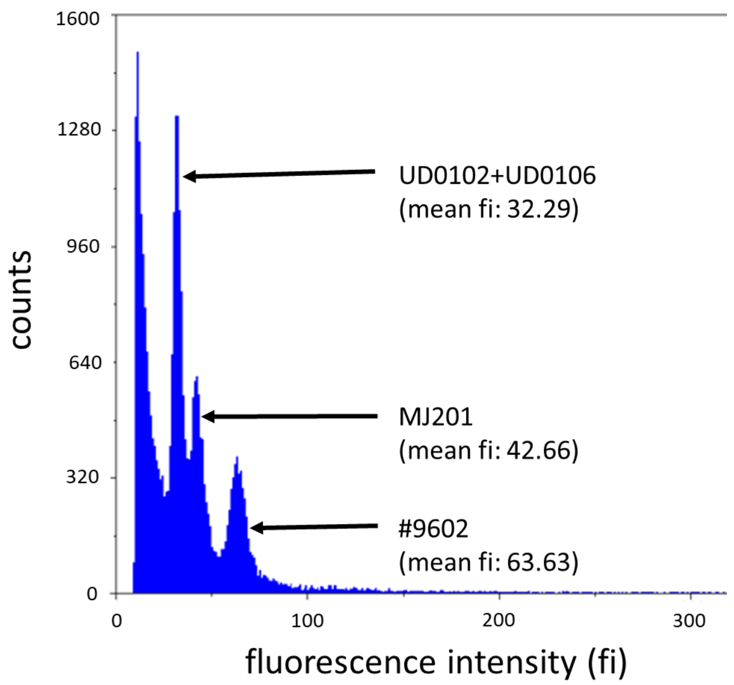

Figure S1. Relative genome size measurement of the investigated clones. The tetraploid clone #9602 shows a ca. twofold fluorescence intensity compared to the diploid clones UD0102 and UD0106.

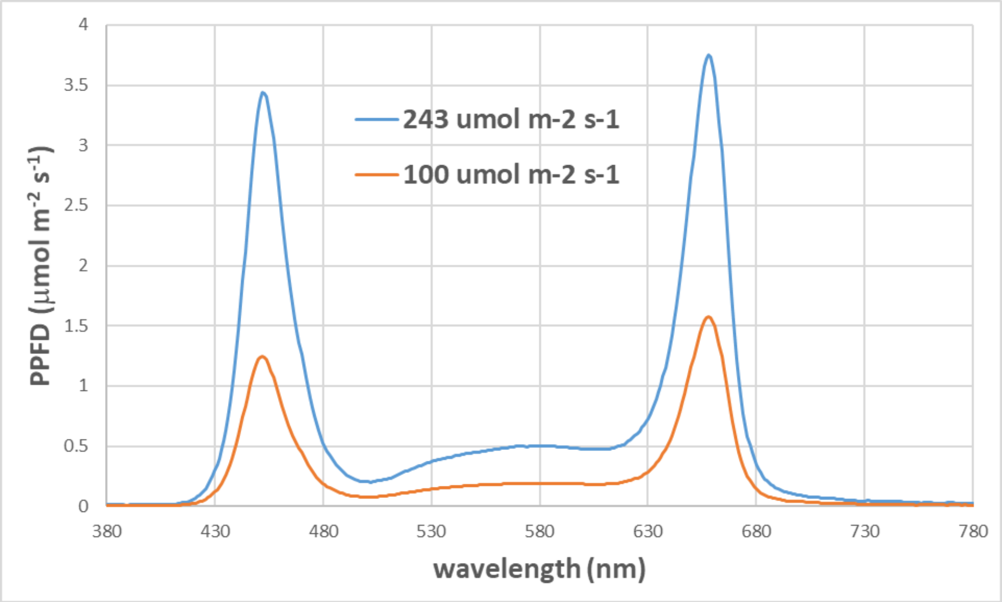

Figure S2. Spectral composition of the light environment used in the “low“ (100 μmol m^{-2} s^{-1}) and “high” light treatments (243 μmol m^{-2} s^{-1}), respectively. The spectra were recorded and averaged (n=5) by means of an MS-100 InSight spectroradiometer (Apogee Instruments Inc., Logan, UT, USA)

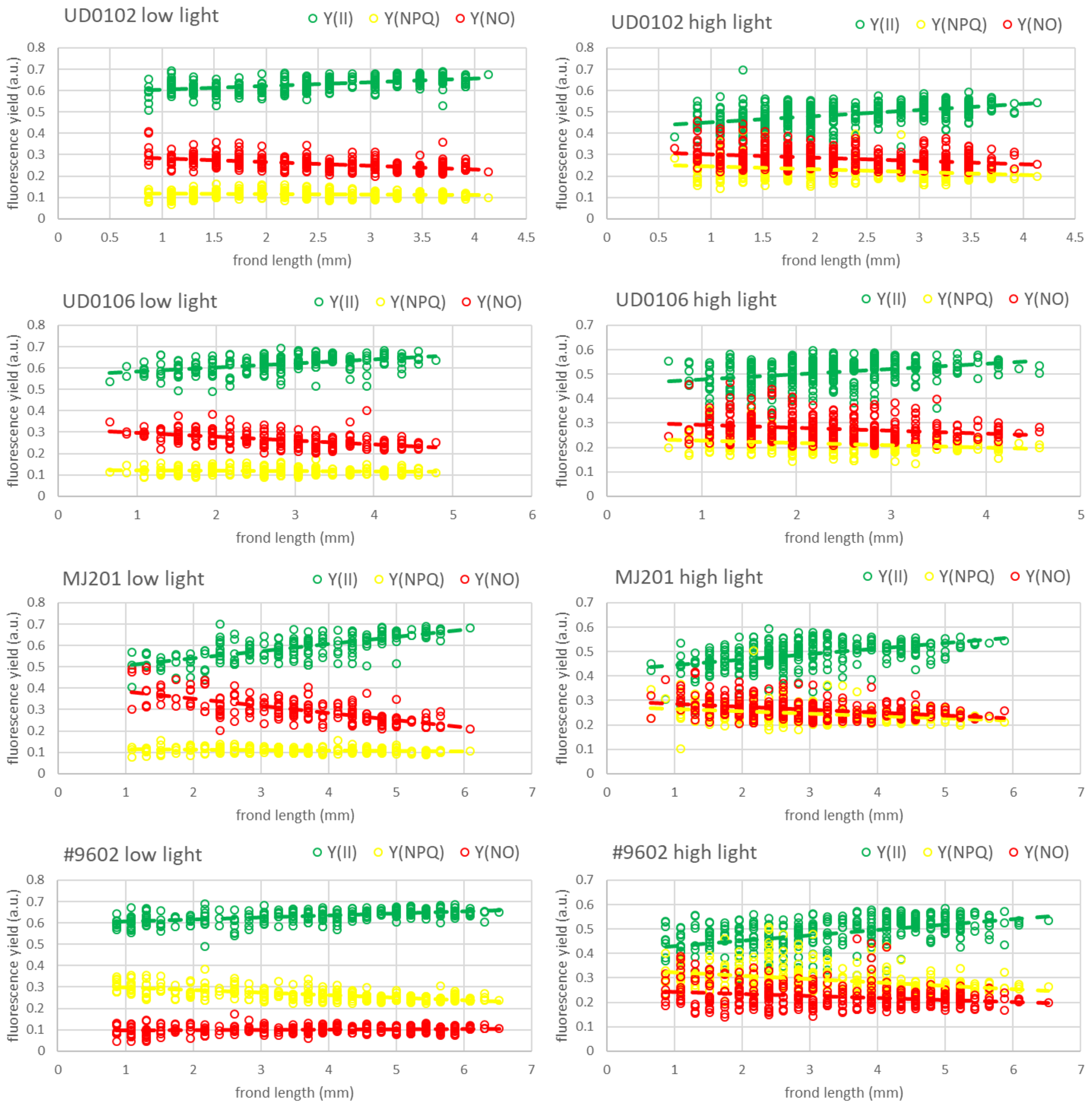

Figure S3. Allocation of absorbed excitation energy in *L. gibba* fronds during their maturation. Y(II) (green) denote photochemical quantum yield (i.e. photosynthesis), while Y(NPQ) (yellow) and Y(NO) (red) denote proportions of regulated and non-regulated photochemical quenching, respectively, as functions of frond length (x-axis). The four *L. gibba* clones (in rows) were cultivated under lower (left column) and higher growing light intensities (right column). Circles denote measurements on individual fronds; the fitted linear regression models are plotted as dashed lines with matching colors.

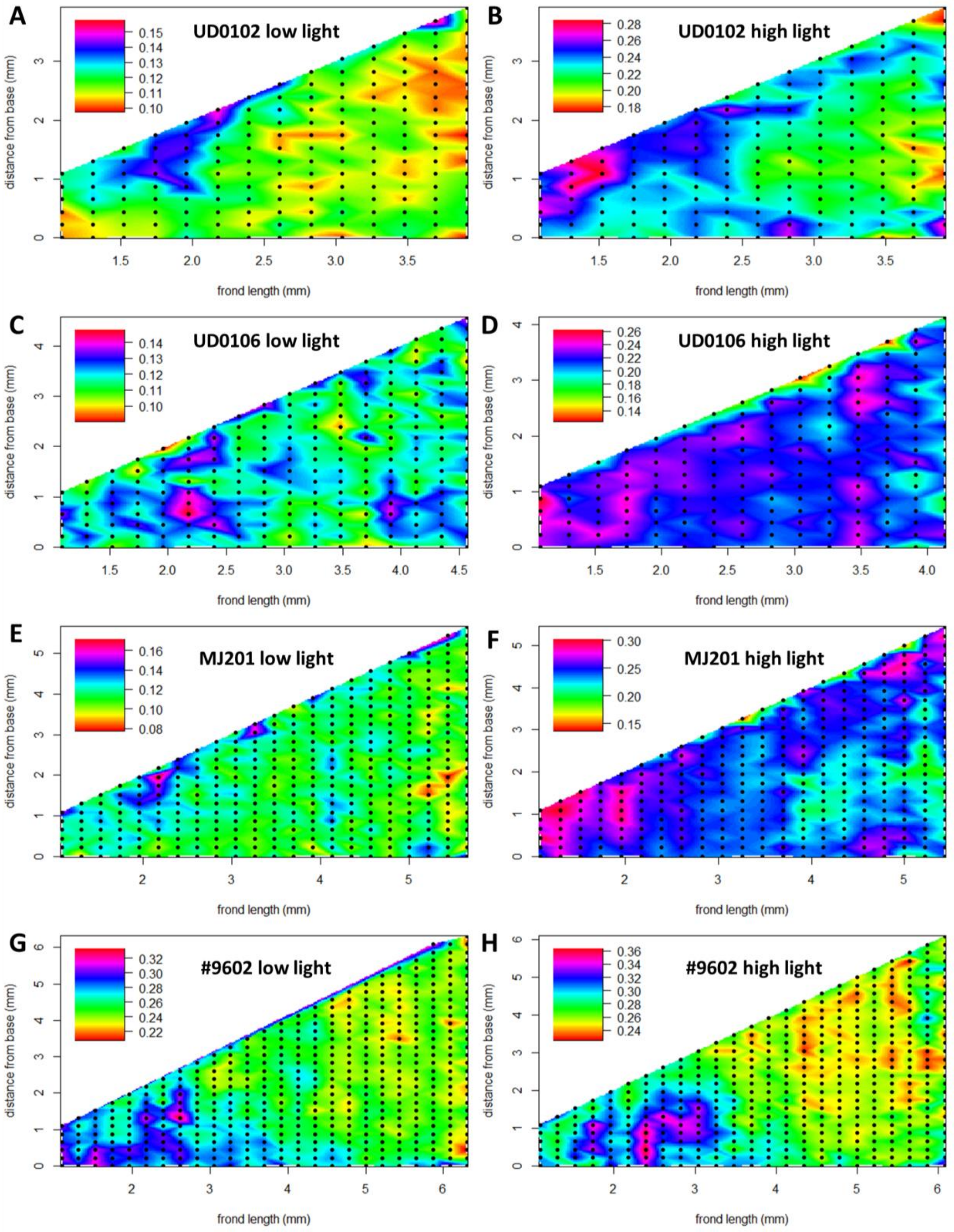

Figure S4. Within-frond patterns in the yield of regulated non-photochemical quenching [Y(NPQ)] during ontogenesis of the studied four L. gibba clones (rows), under low- (left column) and high light treatments (right column), respectively. The interpolation plots are composed of pixel-by-pixel Y(II) values along longitudinal transects in fronds with different sizes. Frond size classes are arranged on the x-axis, while within-frond position measured from the basal tip is plotted on the y-axis. Black dots denote positions of the actually measured pixels along the transects. Note: color gradients are set individually for each plot.

Table S1. Flow cytometric measurements* and calculated relative genome sizes as ratio of fluorescence intensity of the sample and the fluorescence intensity of the internal standard *Lemna aequinoctialis* clone #BOG0001.

	**fluorescence intensities**			**fluorescence intensities**			
**clone**	**sample**	**#BOG0001**	**ratio**	**sample**	**#BOG0001**	**ratio**	**mean ratio**
UD0102	30.33	48.99	0.62	31.43	50.02	0.63	0.62
UD0106	31.38	49.26	0.64	32.02	51.71	0.62	0.63
MJ201	41.83	49.81	0.84	40.84	48.80	0.84	0.84
#9602	62.55	49.80	1.26	60.96	49.26	1.24	1.25
Lemna gibba reference samples from Braglia et al. [~~1~~29] **							
#7796	31.25	50.05	0.62	30.12	48.06	0.63	0.63
#7922	31.04	48.97	0.63	30.42	48.83	0.62	0.63
#9248 (GGM)	41.08	49.41	0.83	38.25	46.65	0.82	0.83
#9425a (GGM)	41.53	49.70	0.84	39.51	47.17	0.84	0.84

*To investigate the relative genome size (RGS) of the investigated clones, we performed flow cytometry using a CyFlow Space flow cytometer (Sysmex Partec GmbH, Görlitz, Germany). To extract nuclei from fresh plant tissue, about 2-3 fully grown fronds of the sample were chopped carefully together with 3-4 fronds of the internal standard *Lemna aequinoctialis* Welw. clone #BOG0001 in 500 µl of Otto I buffer [0.1 M citric acid, 0.5% (v/v) Tween-20; Ulrich and Ulrich, 1991] with a sharp razor blade. After incubation for 2 min on ice and subsequent filtering (ca. 30 µm filter size), 500 µl of the staining Otto II buffer (0.4 M Na_2HPO_4, 4 mg/ml DAPI; [~~2~~68]) were added and the sample was measured after another incubation for 2 min in darkness in the flow cytometer equipped with a 375 nm UV laser. Data collection was stopped after a minimum of 10,000 events, and the relative genome sizes were calculated as the proportion of fluorescence intensities of the sample relative to the internal standard. All samples were measured in replicates.

** For comparison of RGS, reference samples from Braglia et al. [~~1~~29] were measured. A ratio of 0.62-0.63 (UD0102, UD0106) is in the range for the RGS of *Lemna gibba*, while the ratio of 0.84 (MJ201) is in the range of the RGS measurement for the two clones #9248 and #9425a which were identified as triploid hybrids *L.* × *mediterranea* with two portions of *L. gibba* and one portion of *L. minor* genome (GGM) by Braglia et al. [29~~1~~].

~~1~~29. Braglia, L.; Ceschin, S.; Iannelli, M.A.; Bog, M.; Fabriani, M.; Frugis, G.; Gavazzi, F.; Gianì, S.; Mariani, F.; Muzzi, M.; et al. Characterization of the Cryptic Interspecific Hybrid *Lemna×mediterranea* by an Integrated Approach Provides New Insights into Duckweed Diversity. *J. Exp. Bot.* **2024**, *75*, 3092–3110, doi:10.1093/jxb/erae059.

~~2~~68. Ulrich, I.; Ulrich, W. High-Resolution Flow Cytometry of Nuclear DNA in Higher Plants. *Protoplasma* **1991**, *165*, 212–215, doi:10.1007/BF01322292.

Table S2. Parameters of the fitted linear regression models describing trends in photochemical quantum yield (i.e. photosynthesis) - Y(II), regulated non-photochemical quenching - Y(NPQ), and non-regulated non-photochemical quenching - Y(NO) as functions of frond length in the four studied L. gibba clones, UD0102, UD0106, MJ201 and #9602, respectively.

**Y(II)**	slope (a)	intercept (b)	Pearson's r	p (uncorrelated)	value at min frond size	value at max frond size
UD0102 low light	0.0178	0.585	0.512	<0.001	0.603	0.659
UD0102 high light	0.0292	0.423	0.461	<0.001	0.452	0.543
UD0106 low light	0.0190	0.564	0.460	<0.001	0.583	0.655
UD0106 high light	0.0215	0.457	0.312	<0.001	0.478	0.555
MJ201 low light	0.0333	0.475	0.690	<0.001	0.508	0.677
MJ201 high light	0.0226	0.422	0.439	<0.001	0.444	0.554
#9602 low light	0.0094	0.597	0.535	<0.001	0.606	0.658
#9602 high light	0.0226	0.408	0.482	<0.001	0.431	0.556
**Y(NPQ)**	slope (a)	intercept (b)	Pearson's r	p (uncorrelated)	value at min frond size	value at max frond size
UD0102 low light	-0.0022	0.119	-0.128	0.028	0.117	0.110
UD0102 high light	-0.0137	0.260	-0.273	<0.001	0.247	0.204
UD0106 low light	-0.0016	0.122	-0.099	0.153	0.121	0.114
UD0106 high light	-0.0091	0.237	-0.226	<0.001	0.228	0.195
MJ201 low light	-0.0016	0.116	-0.157	0.032	0.114	0.106
MJ201 high light	-0.0097	0.275	-0.290	<0.001	0.265	0.218
#9602 low light	-0.0116	0.308	-0.646	<0.001	0.297	0.233
#9602 high light	-0.0139	0.338	-0.389	<0.001	0.324	0.248
**Y(NO)**	slope (a)	intercept (b)	Pearson's r	p (uncorrelated)	value at min frond size	value at max frond size
UD0102 low light	-0.0179	0.302	-0.521	<0.001	0.284	0.228
UD0102 high light	-0.0160	0.318	-0.313	<0.001	0.302	0.252
UD0106 low light	-0.0176	0.314	-0.467	<0.001	0.297	0.230
UD0106 high light	-0.0114	0.303	-0.188	0.001	0.291	0.251
MJ201 low light	-0.0337	0.420	-0.692	<0.001	0.386	0.215
MJ201 high light	-0.0118	0.298	-0.351	<0.001	0.286	0.229
#9602 low light	-0.0087	0.250	-0.290	<0.001	0.241	0.193
#9602 high light	0.0017	0.095	0.147	0.014	0.096	0.105
